# Evaluating surrogates for overall survival in the adjuvant treatment of bladder cancer with chemotherapy

**DOI:** 10.1016/j.urolonc.2025.07.013

**Published:** 2025-08-21

**Authors:** C. N. Sternberg, P. Squifflet, E. D. Saad, S. Burdett, D. Fisher, M. Kurt, S. Teitsson, J. R. May, M. Patel, M. Stoeckle, F. Torti, R. Cote, E. M. Ruggeri, A. Zhegalik, J. F. Tierney, L. Collette, T. Burzykowski, M. Buyse

**Affiliations:** 1 Englander Institute for Precision Medicine, Weill Cornell Medicine, New York, New York, USA; 2 IDDI, Louvain-la-Neuve, Belgium; 3 MRC Clinical Trials Unit at UCL, London, UK; 4 Bristol Myers Squibb, Princeton, New Jersey, USA; 5 Bristol Myers Squibb, Uxbridge, UK; 6 Department of Urology, Saarland University Medical Center, Homburg, Germany; 7 Department of Medicine, University of Connecticut Health Center, Farmington, Connecticut, USA; 8 Department of Pathology and Immunology, Washington University in St. Louis, St. Louis, Missouri, USA; 9 UOC Oncologia e Rete Oncologica, ASL, Viterbo, Italy; 10 Urology, N. N. Alexandrov National Cancer Center, Lesnoy, Minsk District, Belarus; 11 Data Science Institute, Hasselt University, Diepenbeek, Belgium

**Keywords:** Bladder neoplasms, platinum compounds, adjuvant therapy, surrogate endpoints, overall survival, disease-free survival

## Abstract

**Introduction:**

Surrogates for overall survival (OS) can expedite the development of adjuvant treatments for bladder cancer. We evaluated whether disease-free survival (DFS) or distant metastasis-free survival (DMFS) are valid surrogates for OS in patients with muscle-invasive disease treated with cisplatin-based chemotherapy after radical cystectomy.

**Methods:**

We analyzed individual patient data from 1075 patients enrolled in nine randomized controlled trials (RCTs) identified by systematic review. These RCTs compared adjuvant cisplatin-based chemotherapy combined with local treatment versus local treatment alone and excluded neoadjuvant chemotherapy. We measured the patient-level association between DFS/DMFS and OS using Spearman’s correlation coefficient (ρ), and the trial-level association between hazard ratios (HRs) using R^2^. For both measures, values close to 1.00 are required for surrogate validation. We assessed the intent-to-treat (ITT) populations and subgroups defined by lymph node status.

**Results:**

The evaluation of DFS in the ITT population showed ρ = 0.89 (95% confidence interval [CI] 0.87–0.90) and R^2^ = 0.69 (95% CI 0.34–1.00). Corresponding measures for DMFS were ρ = 0.91 (95% CI, 0.89–0.92) and R^2^ = 0.90 (95% CI, 0.74–1.00). Patient-level associations were moderate or strong regardless of the lymph node status. At the trial level, DFS displayed weak association with OS in lymph node–positive patients, but associations were strong for lymph node–negative patients and for DMFS.

**Conclusion:**

In the adjuvant treatment of bladder cancer with cisplatin-based chemotherapy, DFS is a moderate to strong surrogate for OS, while DMFS is a strong surrogate for OS.

## 1 Introduction

Despite the curative potential of radical cystectomy, up to two-thirds of patients with localized and locally advanced bladder cancer (BC) ultimately develop recurrent or distant metastatic disease with postoperative observation alone [1–3]. Both neoadjuvant and adjuvant cisplatin-based chemotherapy are currently accepted systemic therapy options for eligible patients with muscle-invasive BC (MIBC), based on patient and disease characteristics and institutional preference [4,5]. Although neoadjuvant chemotherapy improves long-term outcomes, use of this treatment modality is not as widespread as would be expected [3,6]. On the other hand, based on its ability to improve disease-free survival (DFS) and overall survival (OS) [5], adjuvant chemotherapy is another viable option for cisplatin-eligible patients with MIBC undergoing surgical resection with no previous neoadjuvant therapy [4].

Recent results with immune checkpoint inhibitors in the adjuvant setting suggest a role for immunotherapy in improving long-term outcomes after surgery [7,8]. In these trials, DFS was the primary endpoint and was significantly improved by the use of adjuvant nivolumab or pembrolizumab among patients with urothelial carcinoma and a high risk of recurrence after radical cystectomy [7,9]. As in other areas in oncology, the use of surrogate endpoints for OS, such as DFS and distant metastasis-free survival (DMFS), may expedite the approval of novel agents for early-stage BC and enable earlier access to these agents without being influenced by the confounding effects of subsequent treatments. Candidate surrogate endpoints should ideally be validated using individual patient data from randomized controlled trials (RCTs) [10]. This type of evaluation should focus on two measures of association between a potential surrogate endpoint (such as DFS or DMFS) and the final endpoint of interest (in this case, OS): (i) the individual-level (or patient-level) association and (ii) the trial-level (or treatment-level) association [10]. The individual-level association denotes the prognostic information provided by the surrogate endpoint—whether patients with prolonged DFS/DMFS are also more likely to experience prolonged OS. The trial-level association provides predictive information on whether treatments that produce changes of a certain magnitude in DFS/DMFS are likely to produce proportional changes in OS. DFS and DMFS have not been evaluated as surrogates for OS on the basis of a systematic review of RCTs of adjuvant, cisplatin-based chemotherapy for BC. The current study was conducted to address this question.

## 2 Methods

### 2.1 Study funding, oversight, and objectives

This study was designed and conducted by the authors, who vouch for the accuracy of the entire work and of the current article. A study protocol for the current work was jointly written by the authors and submitted for approval by the Ethics Committees of Hasselt University, Belgium, and University College London, UK. The project consisted of a post hoc analysis of data from institutional review board–approved RCTs, and as such did not raise any new ethical issues about the collection and processing of clinical data. For all RCTs included in the current analysis, the same data used in a previously published meta-analysis [5] were retrieved after the approval from the original sponsors of each RCT. The protocol for the original project was registered on PROSPERO (CRD42017079637). Costs associated with data collection, management, and analysis were defrayed by financial support provided by the sponsor.

The overarching objective of the study was to assess DFS as a surrogate for OS, primarily for the intent-to-treat (ITT) population of each RCT. Secondary objectives were to assess DMFS as a surrogate for OS and to conduct subgroup analyses for both endpoints at individual and trial levels based on lymph node status. The latter analysis was motivated by the prognostic relevance of lymph node status. Sensitivity analyses were conducted with follow-up truncated at 7 years because some adjuvant RCTs had unusually long follow-up times. This time point was chosen arbitrarily but was deemed sensible from a clinical perspective.

### 2.2 Eligibility of RCTs

The original identification of eligible RCTs took into account the PICOS (Population, Intervention, Comparator, Outcome, Study type) framework [5]. Eligible RCTs (Study type) had compared adjuvant cisplatin-based chemotherapy plus radical cystectomy (Intervention) versus the same local treatment alone (Comparator) in patients with locally advanced BC (excluding upper-tract tumors), were closed to patient accrual, and did not include patients treated with prior neoadjuvant chemotherapy (Population). All endpoints available from each RCT were of interest (Outcome), including OS and particularly those needed to define DFS and DMFS. The RCTs considered eligible for the current study were those previously identified by the systematic review using the above PICOS framework; the search strategy is outlined and the PRISMA flow diagram available as Supplementary Figure 1 in the original publication[5]. A summary is also provided in the supplementary materials to the current article. Of note, the search strategy for the original meta-analysis was the same used here, given the goal of assessing the validity of DFS as a surrogate for OS in this historical set of RCTs with long-term follow-up. Moreover, the search was updated before the current surrogacy work, and no further eligible RCTs were found. A total of 13 RCTs were deemed potentially eligible; 12 were published in full or in abstract form [11–21] and one was unpublished (Omura G: Phase III adjuvant chemotherapy with CTX/ADT/CACP for resected transitional cell bladder carcinoma). Three of the 13 eligible trials were excluded from the analysis due to failure to obtain data from those trials despite attempted contact with investigators [11,17] (Omura G, unpublished data). Moreover, one RCT did not publish or provide data on recurrences and was thus excluded [22], leaving nine RCTs in total to analyze [12–16,18–21].

### 2.3 Statistical methods

The main variables of interest within each eligible trial were sex, age, tumor-node-metastasis indicators, lymph node stage, tumor grade, and dates of randomization, disease recurrence, death, or latest follow-up. All analyses were conducted using SAS, version 9.4 (Cary, NC, USA). A two-tailed level of significance of 0.05 was used throughout, with no formal adjustment for multiplicity and no imputation of missing data (previous knowledge of the trial data indicated a very small amount of missingness). OS was defined as the time between randomization and death from any cause, with censoring of patients who were alive on the last follow-up date. DFS was defined as the time between randomization and any type of recurrence (local or distant) or death from any cause, with censoring of patients who were alive and recurrence-free on the last follow-up date. Since not all RCTs collected data on distant metastasis after locoregional recurrence events, DMFS was defined as the time between randomization and distant recurrence as a first event or death from any cause, with censoring of patients who were alive and distant recurrence-free on the last follow-up date. Thus, for RCTs that recorded only the first recurrence, patients having a locoregional recurrence were censored at the time of locoregional recurrence in the analysis of DMFS.

A standard two-level meta-analytic modeling approach was used to estimate the association between candidate surrogates (DFS and DMFS) and OS, and between the treatment effects on these endpoints [23]. At the patient level, the joint distribution of DFS or DMFS and OS was estimated using three copula-based models (Clayton’s, Hougaard’s, or Plackett’s) as candidates that were assessed by the maximum likelihood value they generated for the data. The strength of the patient-level association between DFS or DMFS and OS was quantified by the value of Spearman’s rank correlation coefficient (ρ). For the trial-level assessment, Cox’s (stratified) proportional-hazards model was used to jointly estimate the hazard ratios (HRs) for DFS (or DMFS) and OS. A linear regression was then fitted through the points representing the logarithms of the hazard ratio (logHR) for DFS (or DMFS) and for OS from each RCT. For simplicity of interpretation, figures depicting the surrogacy relationships have been adapted such that the actual HRs for the endpoints of interest, rather than their logarithmic transformations, are shown on the axes. In quantifying the association between the treatment effects on the candidate surrogate and OS, an attempt was first made to fit regression models that take the estimation error present in the estimated HRs for each endpoint into account using a measurement error model [23]. In case of model non-convergence, weighted linear regression (WLR) models were considered. WLR is a flexible approach to estimating trial-level correlations, especially when adjusted models do not converge. In WLR models, the pair of treatment effects on the surrogate endpoint and on OS for the same RCT represent a data point, and each of these data points can be weighted using the sample size or the number of deaths in each trial to reflect the impact of larger trials on the results; here, we used the number of deaths as weights. For both adjusted and WLR models, the linear regression fitted through the estimated treatment effects provides a coefficient of determination (R^2^), which quantifies the proportion of variance for the treatment effect on OS that is explained by the treatment effect on the candidate surrogate. For interpretation of association measures, it should be noted that ρ ranges from –1.00 to 1.00, whereas R^2^ ranges from 0 to 1.00. While values of ρ and R^2^ closer to 1.00 are required for a strong surrogacy relationship, strengths of association were qualified as weak if ρ < 0.7 or R^2^ < 0.50, moderate if 0.7 ≤ ρ < 0.9 or 0.5 ≤ R^2^ < 0.8, and strong if ρ > 0.9 or R^2^ > 0.80. For the trial-level correlation between the endpoints, a leave-one-out cross-validation strategy was used to assess the predictive accuracy of the corresponding model, where each RCT was left out once from the evidence base and the WLR model was refitted using the remaining RCTs. The resulting surrogacy equation from the newly fitted model was reapplied to estimate the predicted OS HR and a corresponding 95% prediction interval for the left-out RCT and compared with the observed OS HR for that trial. In the same manner, for an external validation of the model, for one of the trials not included in the analyses for which published results on DFS and OS were available, surrogacy equations were applied to reported HR for DFS in an attempt to predict its OS HR [17,24]. Finally, the surrogate threshold effect (STE) was computed for selected scenarios. The STE—the minimum treatment effect on the surrogate required to predict a statistically significant treatment effect on the final endpoint in a future randomized trial—was computed for various expected numbers of deaths in the trial using DFS or DMFS as its primary endpoint [25].

## 3 Results

### 3.1 Characteristics of RCTs

Data were available from 1082 patients from the nine trials analyzed (75% of all patients from the 13 eligible RCTs). The key features of these RCTs are shown in Table 1 [12–16,18–21]. The RCTs were conducted between 1980 and 2014, each accruing between 49 and 284 patients. At the time of analysis, the median follow-up for OS across the nine RCTs ranged from 3.5 to 14.8 years, with a median of 6.4 years across all trials. Seven patients (from two RCTs) were excluded from all analyses due to missing data, leaving 1075 patients overall for the analyses (Table 1).

### 3.2 DFS as a surrogate for OS

**Figure 1:**
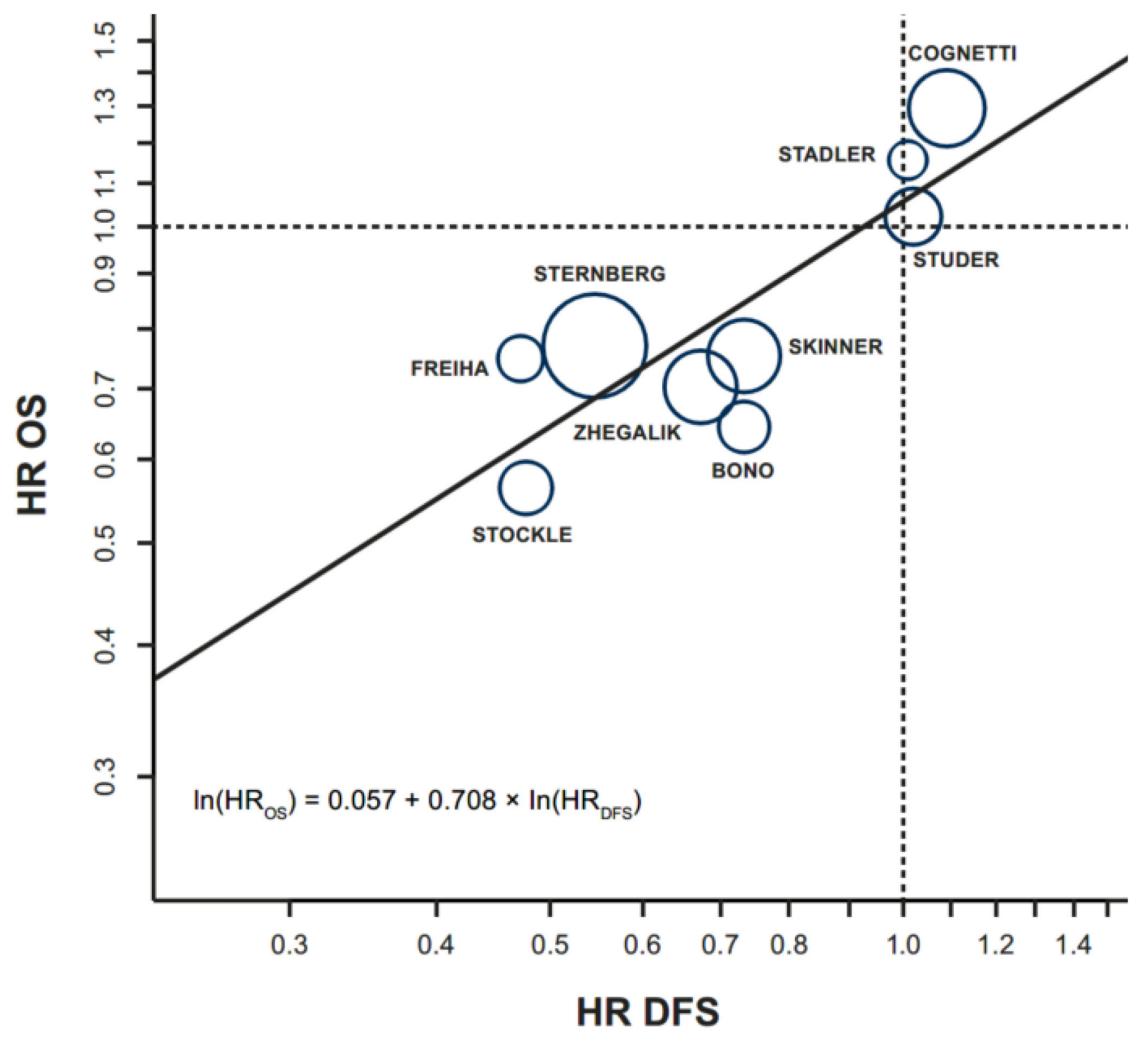
Trial-level association between DFS and OS, intent-to-treat population, weighted linear regression model (each circle represents one trial, with size proportional to the number of deaths). DFS, disease-free survival; HR, hazard ratio; OS, overall survival.

Data for the analysis of DFS were available for all 1075 patients, with 615 DFS events and 553 OS events across the nine RCTs. In the pooled data from all RCTs, the HRs for DFS and OS, stratified by RCT, were 0.70 (95% CI 0.60–0.83) and 0.82 (95% CI 0.70–0.97), respectively. The measures of association are summarized in Table 2. At the patient level, the association between DFS and OS was moderate to strong with little uncertainty, with ρ = 0.89 (95% CI 0.87–0.90). At the trial level, the association was moderate, with R^2^ = 0.69 (95% CI 0.34–1.00) in the WLR model (Figure 1); the equation obtained from the WLR model relating the treatment effects on DFS and on OS (as shown in Figure 1) was ln (HR_OS_) = 0.057 + 0.708 × ln (HR_DFS_). In the regression model adjusting for estimation errors, R^2^ was equal to 0.89 (95% CI 0.00–1.00); this 95% CI was at its maximum width due to high uncertainty around the estimated HRs for DFS and OS from individual RCTs, thus being non-informative. For the sensitivity analysis with respect to follow-up duration, across all RCTs, only 30 DFS events and 36 OS events occurred after 7 years. Therefore, the measures of association with truncation at 7 years were qualitatively similar to those without truncation and are summarized in Table 3.

### 3.3 DMFS as a surrogate for OS

**Figure 2:**
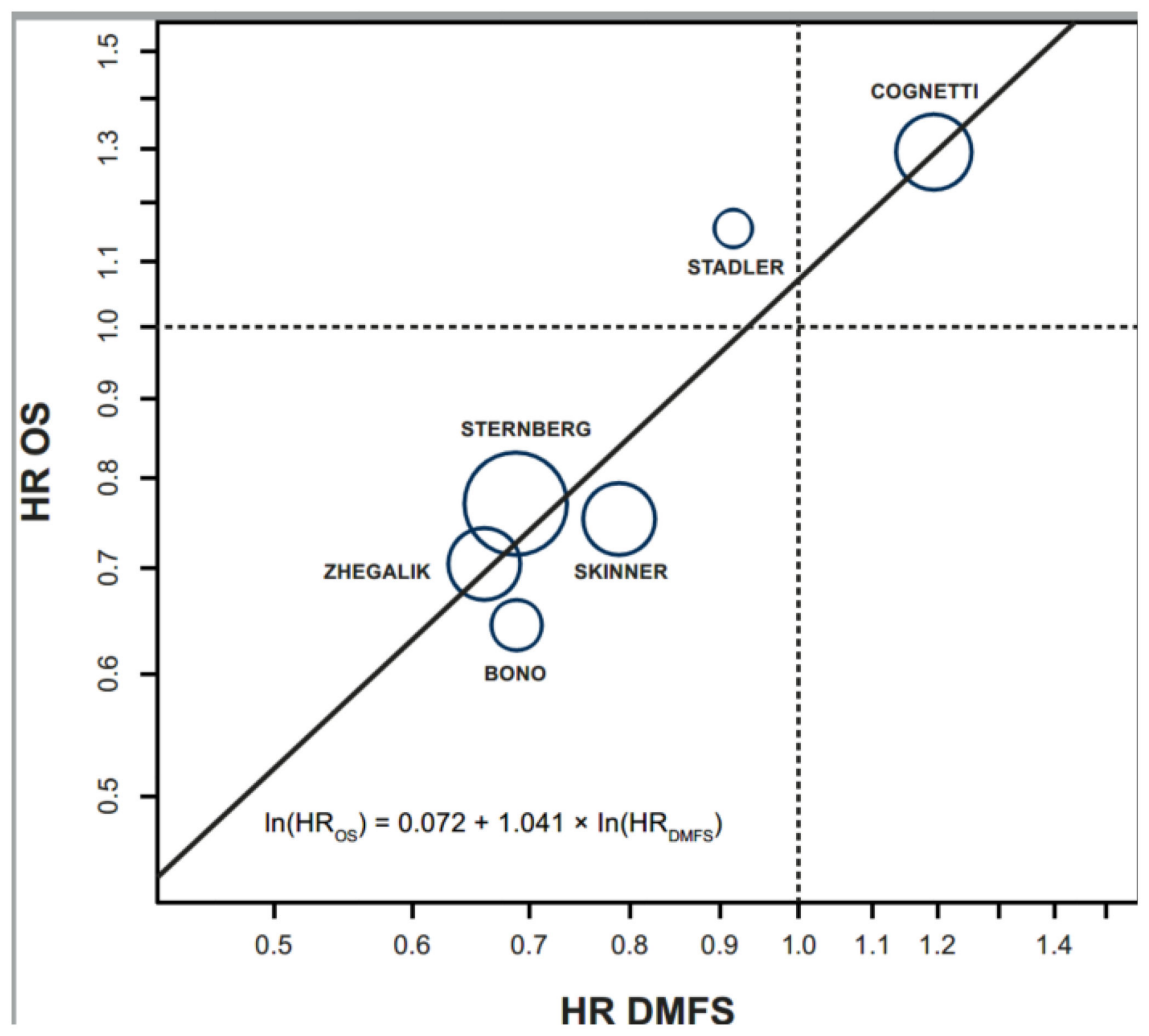
Trial-level association between DMFS and OS, intent-to-treat population, weighted linear regression model (each circle represents one trial, with size proportional to the number of deaths). DFS, disease-free survival; HR, hazard ratio; OS, overall survival.

In this case, data were available for 884 patients from six RCTs providing separate information on distant recurrence, with 474 DMFS events and 438 death events. The pooled HRs for DMFS and OS, stratified by RCT, were 0.80 (95% CI 0.67–0.96) and 0.84 (95% CI 0.69–1.01), respectively. The measures of association are also summarized in Table 2. At the patient level, the association between DMFS and OS was strong with little uncertainty, with ρ = 0.91 (95% CI 0.89–0.92). At the trial level, the WLR model also indicated a strong association, with R^2^ = 0.90 (95% CI 0.74–1.00) (Figure 2). The surrogacy equation relating the treatment effect on DMFS to treatment effect on OS obtained from the WLR model (also shown in Figure 2) was ln (HR_OS_) = 0.072 + 1.041 × ln (HR_DMFS_). The trial-level association between DMFS and OS was also strong in the regression model adjusting for estimation errors, with R^2^ = 0.98 (95% CI 0.47–1.00). For the sensitivity analysis with respect to length of follow-up, across all RCTs, only 24 DFS events and 28 OS events occurred after 7 years. Once again, similar to the results observed for the DFS-OS correlation, results for the DMFS-OS correlation after truncation were qualitatively similar to those without truncation at both individual and trial level, with the caveat that for the ITT population the adjusted model for the trial-level correlation did not converge. The measures of association between DMFS and OS with truncation at 7 years of follow-up are summarized in Table 3.

### 3.4 Subgroup analyses according to lymph node status

Lymph node status was missing from 28 patients (from three RCTs), which were thus excluded from the subgroup analyses. For the lymph node–negative subgroup, the analysis was based on 607 patients (from all RCTs) for DFS and 504 patients (from six RCTs) for DMFS. For the lymph node–positive subgroup, data were available for 440 patients (from seven RCTs) for DFS and 370 patients (from four RCTs) for DMFS. WLR models were fitted to subgroup data. Of note, the adjusted models for the trial-level correlation assessment according to lymph node status did not converge for either of the endpoints. Table 2 displays measures of association without truncation of follow-up at 7 years, and those corresponding to the analyses after truncation are shown in Table 3. Regardless of the lymph node status and truncation at 7 years, patient-level associations between the two candidate surrogate endpoints and OS were moderate or strong. Associations at the trial level were generally strong for both DFS and DMFS regardless of the lymph node status; however, DFS in lymph node–positive patients displayed weak association with OS. These results were almost insensitive to truncation at 7 years.

### 3.5 Cross-validation

Without exceptions, the observed HRs for OS for each left-out RCT were always within the 95% prediction interval of the HR for OS predicted by the surrogacy equations, regardless of surrogate endpoint (DFS or DMFS), truncation of follow-up, or lymph node status (data not shown). The same was true for the external validation of the equations conducted for the trial from Spain, which reported an updated HR of 0.38 both for DFS and for OS (DMFS was not reported) [24]. The surrogacy equation displayed in Figure 1 predicted an HR for OS of 0.53 (95% prediction interval, 0.34-0–83) for this trial, confirming that the observed HR is within the prediction limits. Figures S1 to S4 in the supplementary materials display the results of WLR models for DFS and DMFS without truncation according to lymph node status.

### 3.6 Surrogate threshold effects

Table S1 in the supplementary materials displays STEs according to the expected number of deaths in a hypothetical future trial using DFS or DMFS as primary endpoint, considering the ITT population and without truncation. A technical note is also provided to explain the dependence of the STE on the number of deaths. For relatively small, medium, and large phase III trials using one of these surrogates as primary endpoint, the HR expected to be associated with a significant gain in OS ranges from 0.46 to 0.66 for DFS, and 0.61 to 0.77 for DMFS.

## 4 Discussion

The current study indicates that DFS has strong association with OS at the patient level in patients with MIBC treated with adjuvant cisplatin-based chemotherapy, but only moderate association at the trial level, with predictions subject to relatively high uncertainty (i.e. the measures of association have wide CIs). On the other hand, subgroup analyses suggest that DFS is a strong surrogate for OS among patients with lymph node–negative disease, in whom associations are strong and accompanied by narrow CIs. For DMFS, the study indicates strong patient-level and trial-level associations, with narrower CIs both in the ITT population and in the two subgroups defined by lymph node status.

The current work has potential limitations. Non-convergence of the adjusted models—typically due to the magnitude of estimation errors being similar to or larger than the between-trial variability, often observed when individual RCTs are relatively small—was observed in the case of DMFS and of subgroup analyses by lymph node status. Even though adjusted models are preferable to WLR models—because the former are better able to account for the uncertainty contained in the observed associations between treatment effects—lack of model convergence has been observed previously [26]. In addition to the convergence issue, the study’s evidence base is limited by the relatively small sample sizes of some RCTs, thus being prone to uncertainty due to wide CIs around reported measures of treatment effect on DFS, DMFS, and OS. Particularly for DMFS, the evidence base is limited by the smaller number of trials reporting this outcome, notwithstanding the results that indicate strong associations between this endpoint and OS, and with the caveat that these results only apply to DMFS defined on the first recurrence event.

Despite these limitations, the strengths of patient-level and trial-level associations observed here are similar to those seen in other settings for which a surrogate endpoint for OS has been validated using the commonly employed two-level meta-analytic approach [26–29]. Conversely, there are several examples in the literature of patient-level associations that were found to be strong but were accompanied by trial-level associations that were, at best, moderate [30–34]. Likewise, a recently published study, using a machine-learning approach rather than the meta-analytic approach used here, provides evidence of the patient-level association between time to progression of non-muscle-invasive BC and OS [35]. As explained elsewhere, while patient-level and trial-level associations complement each other in validating a surrogate endpoint, they should be interpreted independently of each other, and the discrepant results between these two levels of assessment observed in other studies can be explained on the basis of statistical reasoning [10]. Nevertheless, clinical or biological mechanisms can arguably underlie differences between settings. For example, most of the successful cases of surrogate validation to date have been in the adjuvant setting, whereas the failed cases were generally in the advanced setting (and in two studies in the neoadjuvant setting, which were also negative [33,36]). The reasons for such findings remain unknown, but one may speculate that the more frequent use of effective salvage therapy in advanced disease and after observation of absence of a pathological complete response after neoadjuvant therapy creates more confounding of the effect of the first treatment on OS. It remains speculative whether this reason can explain the differences in patient-level measures of association between lymph node–negative and lymph node–positive disease observed here.

In conclusion, while acknowledging substantial uncertainty in the observed correlation estimates at both levels, the current study suggests that DFS is a moderate to strong surrogate and DMFS is a strong surrogate for OS in patients with BC receiving adjuvant chemotherapy. While the current results may enable earlier evaluation and design of future trials, similar studies with larger sample sizes are warranted to generalize the findings to other treatment settings, including neoadjuvant and adjuvant therapies with a different mechanism of action such as immunotherapy. Moreover, the impact of salvage treatment for recurrent disease on measures of association should be investigated, as we enter a new era in which patients with metastatic BC have prolonged OS from novel agents [37].

## Supplementary Material

Supplementary Materials

## Figures and Tables

**Table 1:** Key features of the randomized controlled trials analyzed

RCT	Experimental arm	Control arm	Disease stage	No. enrolled (No. analyzed)	Endpoints assessed	DFS events, No.	DMFS events, No.	Deaths, No.
Skinner [12]	Cystectomy + 4 cycles of adjuvant CA + cyclophosphamide	Cystectomy	T3–4, N+, M0	102 (102)	DFS and DMFS	76	75	74
Studer [13]	Cystectomy + 3 cycles of adjuvant C	Cystectomy	T1 (grade2)– T4	91 (91)	DFS	47	NA	45
Stöckle [14]	Cystectomy + 3 cycles of adjuvant MVAC/MVEC	Cystectomy	T3b–T4a	49 (49)	DFS	41	NA	40
Freiha [15]	Cystectomy + 4 cycles of adjuvant CMV	Cystectomy + same CT on relapse	T3b–T4, any N, M0	55 (51)	DFS	34	NA	30
Bono [16]	Cystectomy + 4 cycles adjuvant CM	Cystectomy	T2–4a, N0, M0	93 (90)	DFS and DMFS	41	40	37
Stadler [18]	Cystectomy + 3 cycles of adjuvant MVAC	Cystectomy	pT1/T2N0M0	114 (114)	DFS and DMFS	26	25	21
Cognetti [19]	Cystectomy + 4 cycles of adjuvant GC	Cystectomy + same CT on relapse	pT2G3, pT3– 4, N0–2	194 (194)	DFS and DMFS	103	101	84
Zhegalik [20]	Cystectomy + 2 cycles of adjuvant GC	Cystectomy + same CT on relapse	pT3a–pT4a, and/or N+ M0	100 (100)	DFS and DMFS	74	74	74
Sternberg [21]	Cystectomy + 4 cycles of adjuvant GC, MVAC or high- dose MVAC	Cystectomy + same CT on relapse	pT3–pT4 or pN1–3, M0	284 (284)	DFS and DMFS	173	159	148

A, doxorubicin; C, cisplatin; CT, chemotherapy; DFS, disease-free survival; DMFS, distant metastasis-free survival; E, epirubicin; G, gemcitabine; M, methotrexate; NA, not applicable; V, vinblastine.

**Table 2:** Measures of association between DFS/DMFS and OS

Candidate Surrogate, Subgroup	Trials contributing to analysis, No.	Patients contributing to analysis, No.	Measures of association (95% CI)		
			Individual-level surrogacy (ρ)	Trial-level surrogacy (R^2^)	
				Adjusted model	WLR model
DFS, ITT population	9	1,075	0.89 (0.87–0.90)	0.89 (0.00–1.00)	0.69 (0.34–1.00)
DFS, lymph node negative	9	607	0.93 (0.92–0.95)	No convergence	0.96 (0.91–1.00)
DFS, lymph node positive	7	440	0.82 (0.79–0.85)	No convergence	0.11 (0.00–0.59)
DMFS, ITT population	6	884	0.91 (0.89–0.92)	0.98 (0.47–1.00)	0.90 (0.74–1.00)
DMFS, lymph node negative	6	504	0.94 (0.92–0.95)	No convergence	0.94 (0.82–1.00)
DMFS, lymph node positive	4	370	0.90 (0.88–0.92)	No convergence	0.96 (0.87–1.00)

DFS, disease-free survival; DMFS, distant metastasis-free survival; ITT, intent-to-treat; OS, overall survival; WLR, weighted linear regression.

**Table 3:** Measures of association between DFS/DMFS and OS with follow-up truncation at 7 years

Candidate surrogate, subgroup	Trials contributing to analysis, No.	Patients contributing to analysis, No.	Measures of association (95% CI)		
			Individual-level surrogacy (ρ)	Trial-level surrogacy (R^2^)	
				Adjusted model	WLR model
DFS, ITT population	9	1,075	0.88 (0.86–0.90)	0.86 (0.01–1.00)	0.72 (0.38–1.00)
DFS, lymph node negative	9	607	0.93 (0.91–0.94)	No convergence	0.96 (0.91–1.00)
DFS, lymph node positive	7	440	0.82 (0.79–0.85)	No convergence	0.21 (0.00–0.79)
DMFS, ITT population	6	884	0.90 (0.89–0.91)	No convergence	0.95 (0.86–1.00)
DMFS, lymph node negative	6	504	0.93 (0.91–0.94)	No convergence	0.94 (0.84–1.00)
DMFS, lymph node positive	4	370	0.90 (0.89–0.92)	No convergence	1.00^a^ (0.99–1.00)

DFS, disease-free survival; DMFS, distant metastasis-free survival; ITT, intent-to-treat; OS, overall survival; WLR, weighted linear regression.

a This value has been rounded from 0.998.

## Glossary

BC: bladder cancer
CI: confidence interval
DFS: disease-free survival
DMFS: distant metastasis-free survival
HR: hazard ratio
ITT: intent-to-treat
logHR: logarithm of the hazard ratio
MIBC: muscle-invasive bladder cancer
OS: overall survival
R^2^: coefficient of determination
RCT: randomized controlled trial
STE: surrogate threshold effect
WLR: weighted linear regression
ρ: Spearman’s correlation coefficient
