# Supplementary material for: Evaluating surrogates for overall survival in the adjuvant treatment of bladder cancer with chemotherapy
Source: Urol Oncol. Author manuscript; Available in PMC 2026 Aug 12. (PMC7619355; doi:10.1016/j.urolonc.2025.07.013)
Supplement: Supplementary file 1 [file EMS216241-supplement-Supplementary_Materials.pdf]

Supplement to "Evaluating surrogates for overall survival in the adjuvant treatment of bladder cancer with chemotherapy"

Search Strategy

The search for eligible trials was conducted independently by the Medical Research Council Clinical Trials Unit (MRC CTU, UK) investigators (and reported in full in Advanced Bladder Cancer Meta-analysis Collaborators Group: Adjuvant Chemotherapy for Muscle-invasive Bladder Cancer: a systematic review and meta-analysis of individual participant data from randomised controlled trials. *Eur Urol.* 2022;81:50–61) and by a third party, Pharmerit International (Netherlands). The former was conducted in August 2020, and the latter in February 2019. In both cases, systematic searches were conducted in Medline, Embase, and the Cochrane Central Register of Controlled Trials (CENTRAL). The MRC CTU search also included the following sources: ClinicalTrials.gov, the World Health Organization International Trials Registry Platform, conference proceedings (American Society of Clinical Oncology [ASCO] for 2004–2020; ASCO Genitourinary Conference for 2008–2020; European Cancer Conference for 2004–2020; European Society for Medical Oncology [ESMO] for 2004–2020; American Urology Association [AUA] for 2004–2020; and European Association of Urology [EAU] for 2004–2020), manual searches of the reference lists of all trials identified and relevant review articles, and the direct knowledge of experts in the field. The Pharmerit search included conference proceedings (all 2016–2019) for American Association for Cancer Research, ASCO, AUA, EAU, European Multidisciplinary Congress on Urological Cancers, and ESMO.

Supplementary Figure S1. Trial-level association between DFS and OS, intent-to-treat population, weighted model (each circle represents one trial, with size proportional to the number of deaths). The proportions of the actual sizes of the trials are different than the proportions of the numbers of deaths observed in the trials.

DFS, disease-free survival; HR, hazard ratio; OS, overall survival.

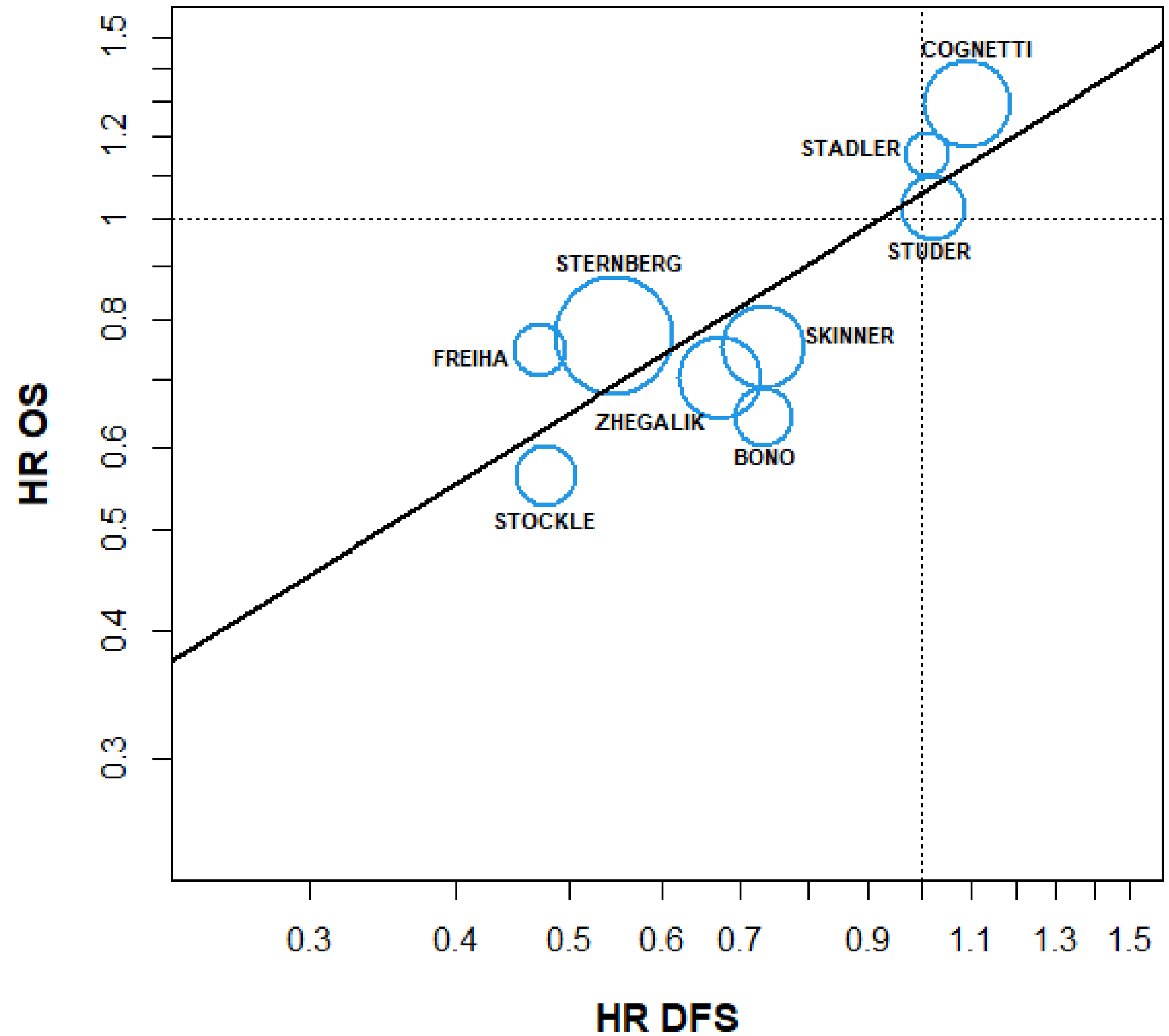
COGNETTI
STADLER
STUDER
STERNBERG
FREIHA
SKINNER
ZHEGALIK
BONO
STOCKLE
HR OS
1.5
1.2
1
0.8
0.6
0.5
0.4
0.3
0.3
0.4
0.5
0.6
0.7
0.9
1.1
1.3
1.5
HR DFS

Supplementary Figure S2. Trial-level association between DMFS and OS, ITT population, weighted model (each circle represents one trial, with size proportional to the number of deaths). The proportions of the actual sizes of the trials are different than the proportions of the numbers of deaths observed in the trials.

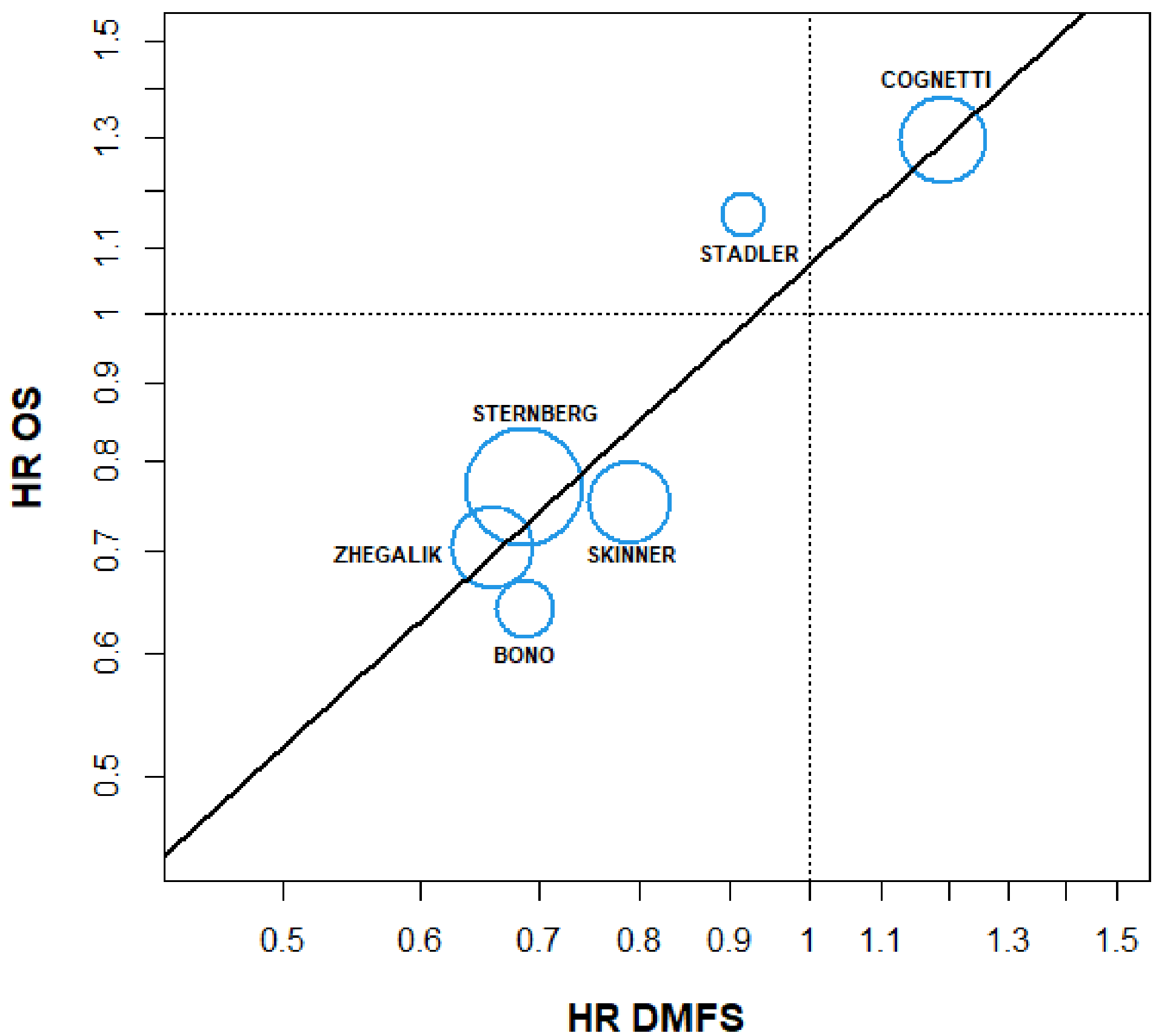

Supplementary Figure S3. Trial-level association between DFS and OS, lymph node–negative patients, weighted model (each circle represents one trial, with size proportional to the number of deaths). The proportions of the actual sizes of the trials are different than the proportions of the numbers of deaths observed in the trials.

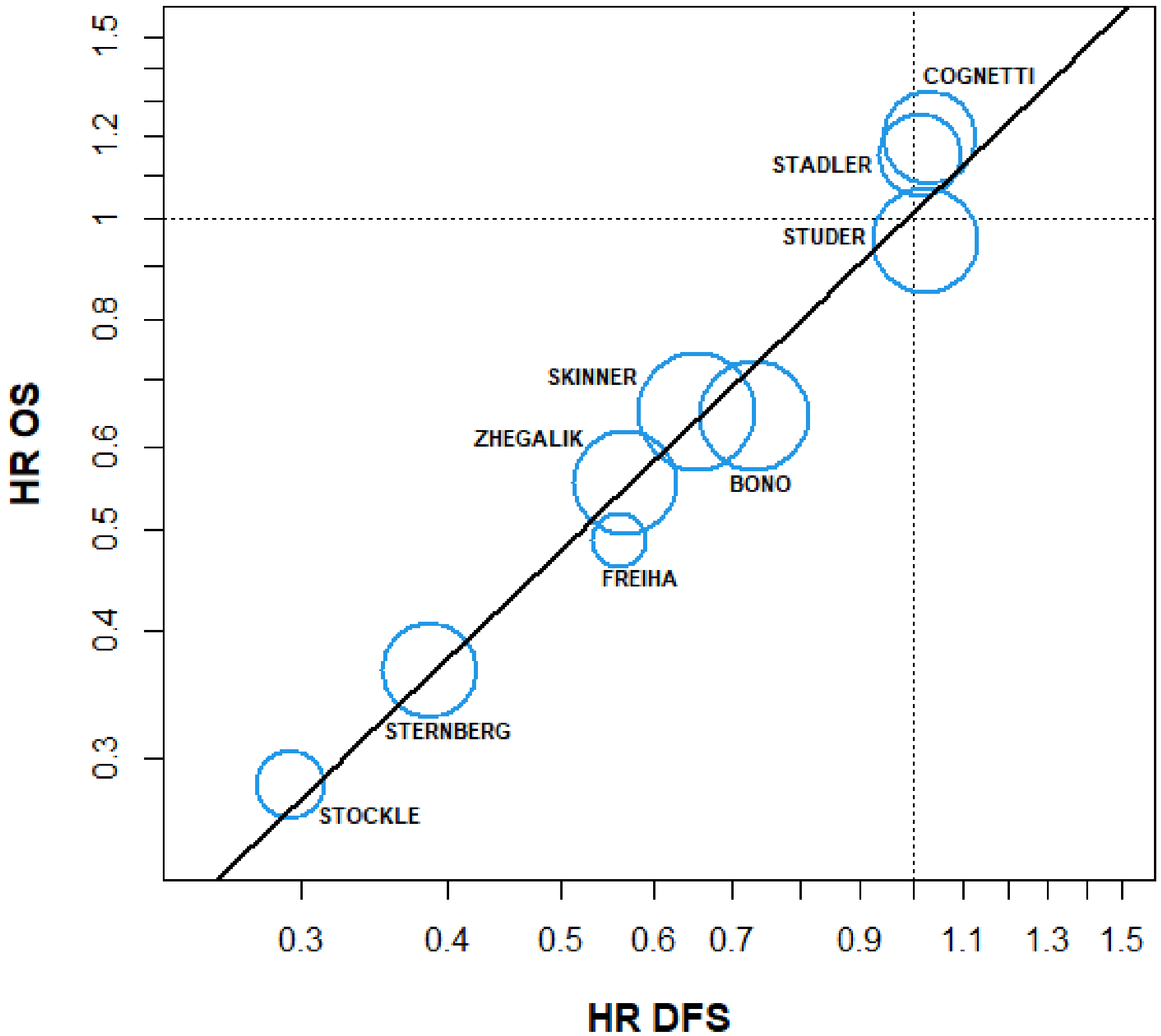

Supplementary Figure S4. Trial-level association between DMFS and OS, lymph node–negative patients, weighted model (each circle represents one trial, with size proportional to the number of deaths). The proportions of the actual sizes of the trials are different than the proportions of the numbers of deaths observed in the trials.

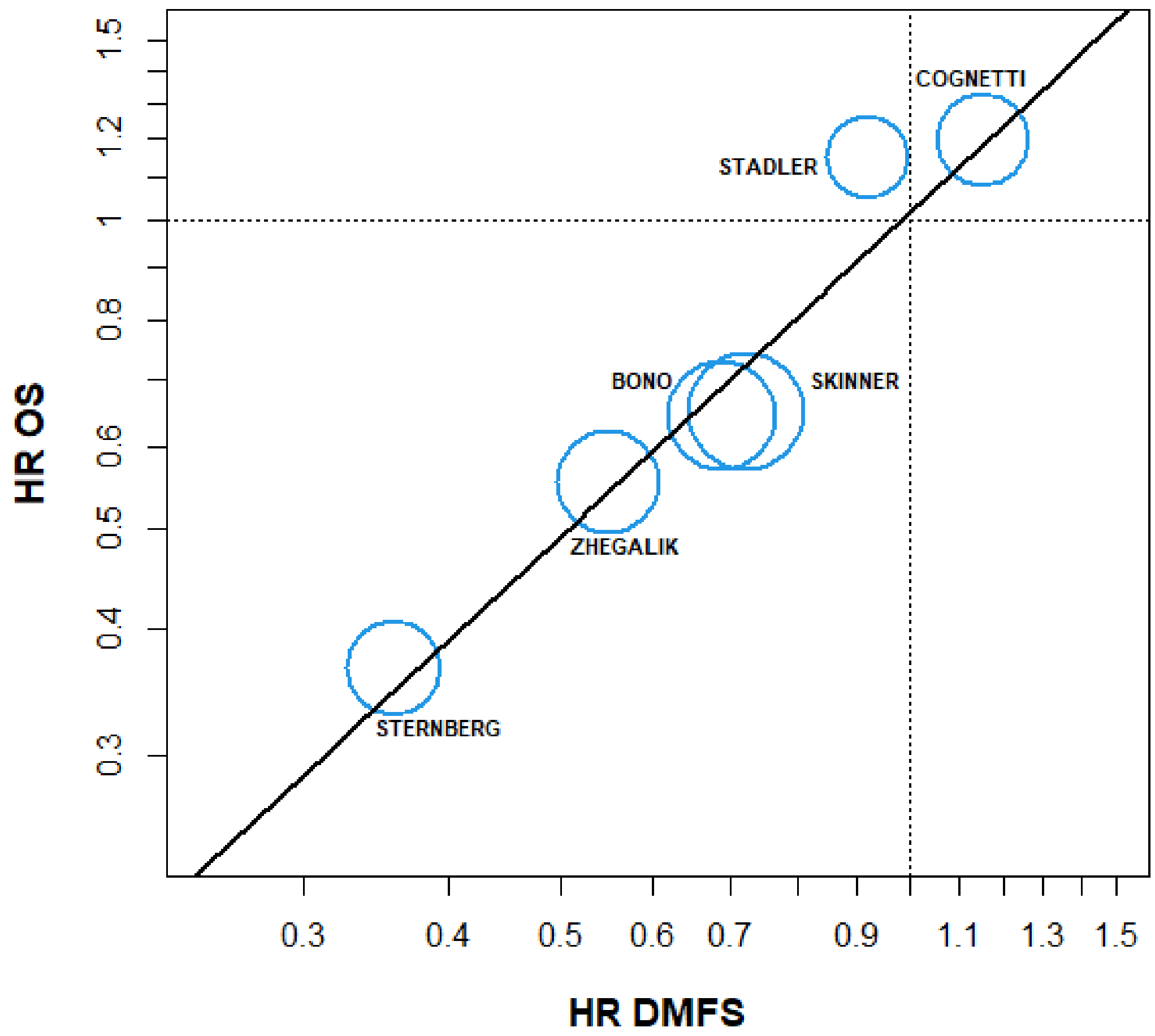

HR OS
1.5
1.2
1
0.8
0.6
0.5
0.4
0.3
COGNETTI
STADLER
BONO
SKINNER
ZHEGALIK
STERNBERG
0.3
0.4
0.5
0.6
0.7
0.9
1.1
1.3
1.5
HR DMFS
